# Tumor-Released Products Promote Bone Marrow-Derived Macrophage Survival and Proliferation

**DOI:** 10.3390/biomedicines9101387

**Published:** 2021-10-04

**Authors:** Juliana Maria Motta, Vivian Mary Rumjanek, Alberto Mantovani, Massimo Locati

**Affiliations:** 1 Instituto de Bioquímica Médica Leopoldo de Meis, Centro de Ciências da Saúde, Universidade Federal do Rio de Janeiro, Rio de Janeiro 21941-902, Brazil; jmotta@bioqmed.ufrj.br (J.M.M.); vivian@bioqmed.ufrj.br (V.M.R.); 2 Humanitas Clinical and Research Center—IRCCS, 20089 Rozzano, Italy; alberto.mantovani@humanitasresearch.it; 3 Department of Medical Biotechnology and Translational Medicine, University of Milan, 20122 Milan, Italy

**Keywords:** macrophage proliferation, tumor-associated macrophages, arginase-1, CD115, CD16

## Abstract

Macrophages play a central role within the tumor microenvironment, with relevant implications for tumor progression. The modulation of their phenotype is one of the mechanisms used by tumors to escape from effective immune responses. This study was designed to analyze the influence of soluble products released by tumors, here represented by the tumor-conditioned media of two tumor cell lines (3LL from Lewis lung carcinoma and MN/MCA from fibrosarcoma), on murine macrophage differentiation and polarization in vitro. Data revealed that tumor-conditioned media stimulated macrophage differentiation but influenced the expression levels of macrophage polarization markers, cytokine production, and microRNAs of relevance for macrophage biology. Interestingly, tumor-derived soluble products supported the survival and proliferation rate of bone marrow precursor cells, an effect observed even with mature macrophages in the presence of M2 but not M1 inducers. Despite presenting low concentrations of macrophage colony-stimulating factor (M-CSF), tumor-conditioned media alone also supported the proliferation of cells to a similar extent as exogenous M-CSF. This effect was only evident in cells positive for the expression of the M-CSF receptor (CD115) and occurred preferentially within the CD16^+^ subset. Blocking CD115 partially reversed the effect on proliferation. These results suggest that tumors release soluble products that not only promote macrophage development from bone marrow precursors but also stimulate the proliferation of cells with specific phenotypes that could support protumoral functions.

## 1. Introduction

Macrophages are widely distributed around tissues and play essential roles in the response to pathogens, maintenance of tissue homeostasis, inflammation, and immunity. They originate from bone marrow myeloid precursor cells, and monocytes are their precursors within the peripheral blood [1]. They are dynamic cells that present different functional profiles according to the stimuli received. Two major classes of macrophages have been described: the classically activated M1 subtype and the alternatively activated M2 subtype [2]. The microenvironment where they are present is paramount for defining their functional and phenotypic profile at specific time points, which can easily be switched according to new environment signals.

Bacterial cell wall components such as lipopolysaccharides (LPS), intracellular pathogens and cytokines like interferon (IFN)-γ, tumor necrosis factor (TNF)-α, and granulocyte-macrophage colony-stimulating factor (GM-CSF) can induce M1 polarization. M1 cells produce high levels of proinflammatory cytokines such as interleukin (IL)-1, IL-6, IL-12, and IL-23, and oxygen radicals. They express high levels of inducible nitric oxide synthase (Nos2), which convert arginine into the cytotoxic compound nitric oxide (NO), and high levels of major histocompatibility complex (MHC) classes I and II [3,4,5,6]. These features provide effective pathogen-killing activity by M1 cells [7]. On the other hand, M2 polarization can be induced by many parasites, fungi, apoptotic bodies, complement proteins, glucocorticoids, and cytokines such as IL-4, IL-13, IL-10, and transforming growth factor (TGF)-β [5]. M2 macrophages secrete less inflammatory cytokines but produce more IL-10. They express high levels of arginase 1 (Arg1), an enzyme responsible for converting arginine into polyamines, thus promoting cell proliferation and fibrosis. They also upregulate the expression of some genes related to tissue remodeling, such as found in inflammatory zone 1 (Fizz1) [4,5,8,9,10,11]. In terms of inflammation, M2 cells act to solve and finalize the inflammatory response; therefore, they also produce extracellular matrix components and angiogenic factors [12]. It has been reported that M2 cells actively participate in organ morphogenesis and tissue turnover, even in non-inflamed sites [13,14]. Furthermore, several subdivisions among M2 macrophages have been described (M2a, M2b, M2c, and M2d) based on differences in cell function.

Diverse authors have published studies on tumor-derived soluble products and their suppressive effects on myeloid immune cell functions in both human and animal models. The secretion of factors, including prostaglandins, IL-6, IL-10, vascular endothelial growth factor (VEGF), and soluble CD44, as well as products from cell metabolism such as succinate, contributes to the immunosuppression found within the tumor microenvironment [15,16,17,18,19,20]. Macrophages play an important role in tumor progression, with relevant implications for metastasis promotion [21,22]. Several studies have reported that the number of macrophages in the microenvironment of human cancers, including breast, prostate, ovarian, and cervical cancer, lung carcinoma, and melanoma, is directly correlated with poor prognosis [23,24]. These macrophages are known as tumor-associated macrophages (TAMs) and they usually share some characteristics with the M2 phenotype (mainly M2d) [25,26,27,28]. Despite the cytotoxic potential of TAMs in certain conditions to provide tumor control (similar to M1 cells), in many other descriptions TAMs have been related to tumor growth, metastasis, angiogenesis, and a reduction in an effective antitumoral immune response [29]. Given the relevance of TAM functions for tumor progression, we aimed to investigate the in vitro effects of tumor-secreted products regarding bone marrow hematopoietic precursor differentiation toward macrophages and their polarization into M1 and M2-like cells, with a focus on their phenotypic features, microRNA expression, and potential for proliferation.

## 2. Materials and Methods

### 2.1. Tumor Cell Lines

3LL and MN/MCA cells were maintained in RPMI 1640 supplemented with 10% heat-inactivated fetal bovine serum (FBS), 100 U/mL penicillin/streptomycin, and 2 mM L-glutamine (all from Lonza, Basel, Switzerland), and split twice a week. Conditioned media (CM) were collected after 3 days of culture, filtered in 0.22 µm filters, and kept frozen at −80 °C. Tumor cells were counted in every passage using Trypan blue (Lonza, Basel, Switzerland) and only 3LL and MN/MCA cultures exhibiting <2% late apoptotic/necrotic cell rates were used to collect CM.

### 2.2. Macrophage Differentiation and Polarization

Bone marrow cells were obtained from femur and tibia of 8–12-week-old C57BL/6J male mice (day 0). Cells were treated with ACK lysis buffer (Thermo Fisher Scientific, Waltham, MA, USA) for 2 min and were cultured in IMDM (Lonza, Basel, Switzerland) supplemented with 10% FBS. Afterwards, cells in suspension (hematopoietic pull) were removed and cultured in a 6-well plate at 0.5 × 10^6^/mL in 3 mL of complete medium (day 1). The hematopoietic precursors were stimulated with 10 ng/mL macrophage colony-stimulating factor (M-CSF) (R&D Systems, Minneapolis, MN, USA) in the presence or absence of a 30% final volume of 3LL CM or MN/MCA CM (day 1). Control points with cells without any stimulation were established, and for all points without CM cells were cultured with 30% RPMI and 70% IMDM to mimic the mix that we needed for CM cultures. On day 4, the medium and stimuli were replaced with the same concentration. On day 6, some cells were treated for a further 24 h with 20 ng/mL IFN-γ (R&D Systems, MN, USA) plus 100 ng/mL LPS from *E. coli* (serotype 055:B5; Sigma Aldrich, MO, USA) to induce the M1 phenotype, or 20 ng/mL IL-4 (R&D Systems, MN, USA) to induce M2. The stimulation done on day 6 was performed without replacement of culture medium. Of note, cells from each animal were separated and treated with the different testing conditions; no mixing of samples from different animals took place.

**Figure 1:**
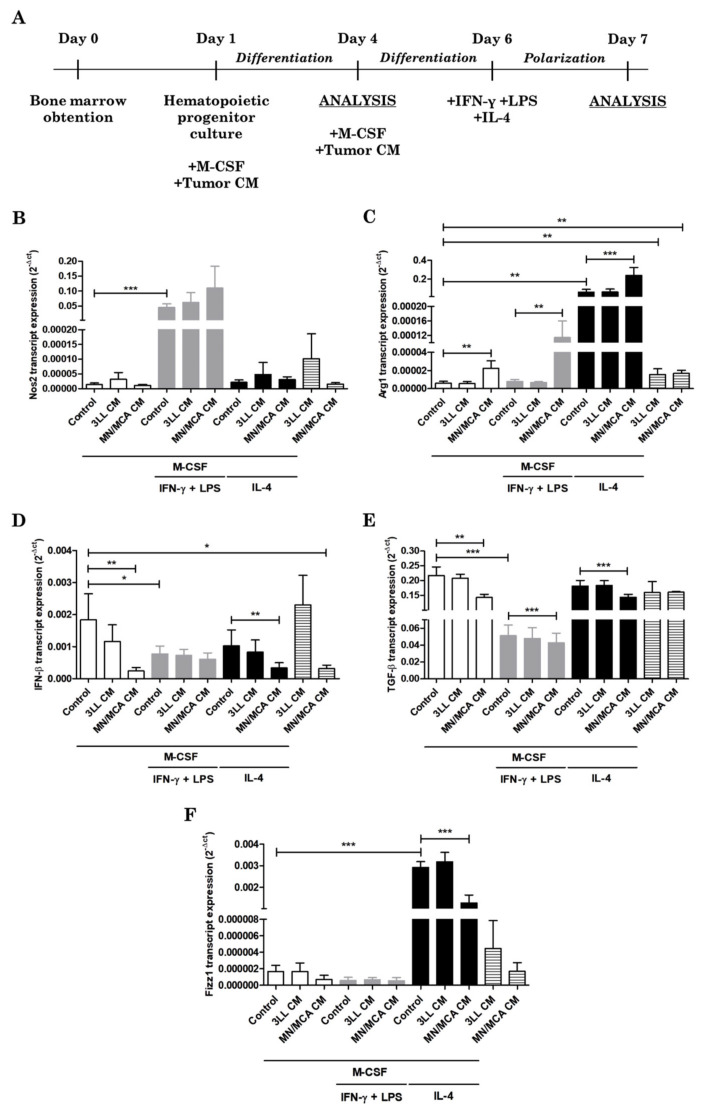
Tumor-conditioned media affect macrophage polarization. (**A**) Protocol of differentiation and polarization of macrophages with tumor products stimulation. (**B**–**F**) Bone marrow hematopoietic progenitor cells were collected and stimulated with M-CSF for 5 days in the presence of 3LL or MN/MCA-conditioned media (CM). After the initial differentiation with M-CSF, IFN-γ plus LPS or IL-4 were added to cultures for a further 24 h to induce polarization. Graphs show the abundance of transcripts encoding inducible nitric oxide synthase (Nos2; (**B**)), arginase 1 (Arg1; (**C**)), interferon-β (IFN-β; (**D**)), transforming growth factor-β (TGF-β; (**E**)), and found in inflammatory zone 1 (Fizz1; (**F**)) in M0 (white bars), M1-like (grey bars), M2-like (black bars), and CM alone (striped bars)-stimulated cells. Results are shown as 2^−Δct^. *n* ≥ 4 independent experiments. *: *p* ≤ 0.05; **: *p* ≤ 0.01; ***: *p* ≤ 0.001.

The Figure 1A summarizes this ex vivo protocol which was approved by the Italian Ministry of Health (6B2B3.N.1CH). All the experiments were conducted in Italy.

### 2.3. Phenotypical Analysis

Macrophages obtained at day 7 were detached from the plate using Accutase (Sigma Aldrich, St. Louis, MO, USA), washed, and fixed with formaldehyde (Sigma Aldrich, MO, USA) for 15 min. Staining was performed using an anti-mouse F4/80-PE, CD206-FITC and MHC II-APC antibodies (R&D Systems, MN, USA). Cells were evaluated in a FACS Canto II flow cytometer and analyzed using FlowJo software. Unstained cells were used as negative control to set auto-fluorescence.

### 2.4. Proliferation Assay

**Figure 3:**
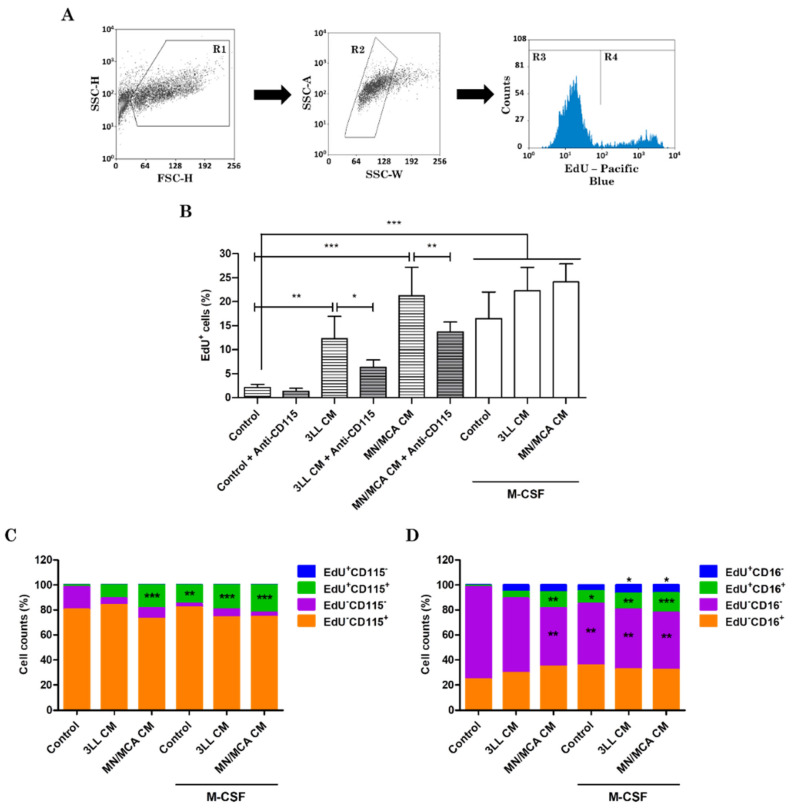
Tumor-conditioned media promote macrophage survival and proliferation. Bone marrow hematopoietic progenitor cells were collected and stimulated with M-CSF in the presence of 3LL or MN/MCA-conditioned media (CM) for 3 days. To some cultures PLX-3397 was also added. (**A**) Flow cytometry strategy to select the populations according to FSC x SSC firstly (left panel), then to exclude cell doublets (middle panel), and finally to separate non-proliferating (R3) and proliferating (R4) groups of cells (right panel). (**B**) On day 4 of culture, cells were stained with an EdU probe and analyzed by flow cytometry. (**C,D**) On day 4 of culture, cells were indeed stained with anti-CD115 or anti-CD16 antibodies to analyze proliferating and non-proliferating groups using flow cytometry. Graphs show EdU^+^ (blue and green) and EdU^−^ (purple and orange) cells investigated for CD115 (green and orange; panel **C**) or CD16 (green and orange; panel **D**) expression. *n* ≥ 3 independent experiments. *: *p* ≤ 0.05; **: *p* ≤ 0.01; ***: *p* ≤ 0.001.

The cell proliferation index was evaluated in the middle (day 4) or on completion of the differentiation/polarization process (day 7). Macrophages were incubated with the EdU probe (Molecular Probes, OR, USA) for 2 h, fixed, and stained with an anti-Pacific Blue secondary antibody (R&D Systems, MN, USA). In some experiments, anti-mouse CD16-APC (R&D Systems, MN, USA) and anti-mouse CD115-PE (R&D Systems, MN, USA) were used along with EdU to characterize the proliferating population. Unstained cells were used as a negative control to set auto-fluorescence, and the gating strategy is shown in Figure 3A. These analyses were performed in a FACS Canto II flow cytometer and analyzed using FlowJo software. M-CSF receptor signaling activity was inhibited using 100 nM Pexidartinib (PLX-3397; MedChemExpress, Monmouth Junction, NJ, USA).

### 2.5. Quantification of Micro and Coding RNA Levels

Relative changes in gene expression were evaluated by quantitative polymerase chain reactions (qPCR) on total RNA isolated using TRIzol (Ambion, Austin, TX, USA). For microRNA analysis, 300 ng of total RNA were reversed transcribed using the TaqMan MiRNA Reverse Transcription kit (Applied Biosystems, Beverly, MA, USA), as previously described [30]. For coding transcripts, 1 µg of total RNA was reversed transcribed using the SuperScriptTM III (Life Technologies, Carlsbad, CA, USA) in accordance with the manufacturer’s instructions. Three replicates per each experimental point were performed. CT values were processed following the 2^−ΔCT^ method, with Gapdh and U6 serving as housekeeping genes. qPCR was conducted using TaqMan probes (mmu-miR-155-5p: ID assay 002571; mmu-miR-511-5p: ID assay 001111; RNU6B (U6): ID assay 001093; Nos2: ID assay Mm00440502_m1; Arg1: ID assay Mm00475988_m1; IFN-β: ID assay Mm00439552_s1; TGF-β1: ID assay Mm01178820_m1; Retnla (Fizz1): ID assay Mm00445110_g1; IL-6: ID assay Mm00446190_m1; IL-10: ID assay Mm01288386_m1; Gapdh: ID assay Mm99999915_g1; Applied Biosystems, MA, USA) in a 7900HT real-time PCR system.

### 2.6. M-CSF and TNF-α Quantification by ELISA

M-CSF (R&D Systems, MN, USA) was measured in 3 samples (collected after 3 different days of culture) of each tumor CM (3LL or MN/MCA), and TNF-α (R&D Systems, MN, USA) was quantified in supernatants collected after macrophage differentiation and polarization (day 7) by enzyme-linked immunosorbent assay (ELISA) exactly according to manufacturer’s instructions.

### 2.7. Statistical Analysis

Statistical analyses were performed using 1-way or 2-way paired ANOVA tests with the Bonferroni post-test in GraphPad Prism software.

## 3. Results

### 3.1. Tumor-Conditioned Media Stimulated Macrophage Differentiation but Affected the Polarization

We first investigated the effect of two types of tumor-conditioned media on macrophage differentiation. For this, the CM of Lewis lung carcinoma and fibrosarcoma tumor cell lines (3LL and MN/MCA, respectively) were added to hematopoietic bone marrow progenitor cultures on day 1 along with M-CSF treatment, and replaced on day 4. On day 6, IFN-α and LPS were added to induce M1-like polarization or IL-4 to M2-like polarization in some cultures (Figure 1A). As shown in Table 1, more than 90% of cells expressed the macrophage marker of differentiation F4/80 on their surface, even in conditions where exogenous M-CSF was not added (tumor CM stimulation alone). Of note, on day 7 we were unable to analyze cells without any stimulation since they did not survive in culture. MHC II surface expression was upregulated in M1-like cells and CD206 in M2-like cells, but tumor CM were not able to affect the expression of these markers (data not shown). Due to this result concerning the macrophage development under the influence of tumor CM alone, the next step was designed to investigate the presence of M-CSF among tumor-secreted products. Therefore, M-CSF was evaluated by ELISA in 3 samples of each tumor CM collected after 3 days of culture in different weeks. We found 19.27 pg/mL of M-CSF in 1 out of 3 3LL CM analyzed and 27.32 ± 5.12 (media ± SD) in all 3 MN/MCA CM.

Following activation/polarization, the two pathways related to L-arginine metabolism were evaluated. The known M1-inducing combination of IFN-γ and LPS induced the expression of Nos2, but no stimulation of Nos2 by tumor CM was observed (Figure 1B). In contrast, the M2 marker Arg1, as expected, was greatly enhanced in all M2-like cells. Nevertheless, MN/MCA CM significantly increased the expression of Arg1, not only in M2-like cells by potentiating M2 inducers, but also in non-polarized (M0) and M1-like macrophages (Figure 1C). Of note, 3LL CM was ineffective in cells stimulated for differentiation with M-CSF, but both tumor CM alone were capable of slightly increasing Arg1 mRNA expression (Figure 1C).

Additionally, IFN-β, TGF-β, IL-6, and IL-10 mRNA expression was evaluated in cells differentiated and polarized in the presence of 3LL and MN/MCA CM. These data revealed that IFN-β expression was not differentially regulated between M1 and M2-like cells; however, MN/MCA CM reduced its expression in M0 and M2-like cells as well as in cells without M-CSF (Figure 1D). When TGF-β was analyzed, we could observe a drastic reduction in its expression by M1-like cells (Figure 1E). In this case, MN/MCA CM was effective in diminishing TGF-β in all conditions in which M-CSF was added (Figure 1E). Regarding IL-6 and IL-10 mRNA expression and TNF-α secretion, no relevant alterations were verified (data not shown). Interestingly Fizz1 mRNA expression was reduced in M2-like cells cultured with MN/MCA CM (Figure 1F).

### 3.2. Macrophage MicroRNAs Are Modulated by Tumor-Conditioned Media

**Figure 2:**
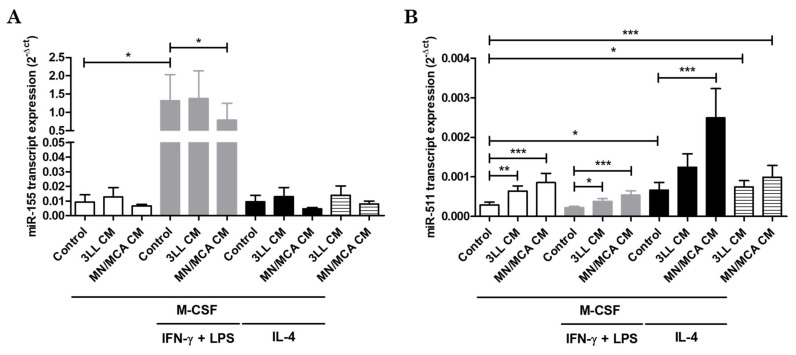
miR-155 and miR-511 expression is modulated by tumor-conditioned media. Bone marrow hematopoietic progenitor cells were collected and stimulated with M-CSF for 5 days in the presence of 3LL or MN/MCA-conditioned media (CM). After this period, IFN-γ and LPS or IL-4 were added to cultures for a further 24 h to induce polarization. Graphs show the abundance of transcripts encoding miR-155 (**A**) and miR-511 (**B**) by M0 (white bars), M1 (grey bars), M2 (black bars), and CM alone (striped bars)-stimulated cells. Results are shown as 2^−Δct^. *n* ≥ 3 independent experiments. *: *p* ≤ 0.05; **: *p* ≤ 0.01; ***: *p* ≤ 0.001.

Macrophage polarization is also associated with changes in the expression levels of several miRs, some with relevant implications for TAM functions [31]. To investigate effects of tumor products on miRs, we specifically analyzed miR-155 and miR-511, which are highly expressed by M1 and TAMs, respectively [32,33]. Our data revealed that MN/MCA CM reduced miR-155 expression in M1-like cells (Figure 2A) but increased miR-511 in all analyzed conditions (M0, M1-like, M2-like, and without M-CSF) (Figure 2B). Conversely, 3LL CM was inactive with regard to miR-155 expression but was also able to induce a higher expression of miR-511 in M0 and M1-like cells and cells without M-CSF stimulation (Figure 2B).

### 3.3. Tumor-Conditioned Media Alone Induce Macrophage Survival and Proliferation

Hematopoietic progenitor cells usually require growth factors to survive in culture for long days [34]. In our experimental setting, unstimulated cells (without M-CSF and/or tumor CM) did not survive until day 7, but tumor CM were capable not only of supporting cell survival but also of promoting cell proliferation even in the absence of M-CSF. When fully differentiated macrophages (day 7) were investigated, the CM proliferative effect was limited and restricted to M0 and M2-like macrophages (around 5%; data not shown), but when cells were investigated at an earlier time point (day 4), this effect became evident and reached levels comparable to those observed in cultures with exogenous M-CSF (Figure 3B). In this case, on day 4 unstimulated cells could be analyzed. When the M-CSF receptor (CD115) signaling activity was blocked using 100 nM PLX-3397, a significant reduction was shown in tumor CM-stimulated cells (53.82% of reversion in 3LL CM and 40.89% in MN/MCA CM) (Figure 3B).

Next, we carried out experiments to analyze the profile of proliferating and non-proliferating cells based on CD115 and CD16 expression. CD16 surface expression characterizes distinct subpopulations of monocytes but has not been deeply investigated in macrophages. When analyzing the expression levels of CD115 on the plasma membrane of macrophages, we could observe that CM induced the proliferation only of cells positive for the expression of CD115. However, independent of proliferation, more than 90% of cells cultured with CM and/or M-CSF expressed CD115, and no impact on the ratio CD115^+^/CD115^−^ could be visualized (Figure 3C). Despite M-CSF being present in some tumor CM analyzed, its concentration was very low or absent in some 3LL CM. Therefore, the role of M-CSF in CM activity may be considered limited. On the other hand, in all conditions tested, cells undergoing proliferation were mostly CD16^+^ (Figure 3D) and this percentage was increased in all analyses compared to unstimulated cells, except for 3LL CM alone. Moreover, both M-CSF 3LL CM and M-CSF MN/MCA CM slightly but significantly enhanced the population EdU^+^CD16^−^ (Figure 3D). Notwithstanding, among proliferating cells the CD16^+^/CD16^−^ ratios observed were between 1.24 (3LL CM alone) and 4.35 (M-CSF MN/MCA CM), whereas all the CD16^+^/CD16^−^ ratios obtained in the non-proliferating group were under 1.0. This suggests that tumor-secreted products may support the proliferation of macrophages undergoing differentiation, preferentially of the CD16^+^ group, which is consistent with data from literature showing that this population is increased in the tumor infiltrate and in the circulation of patients [35,36].

## 4. Discussion

Macrophages constitute an essential part of the tumor mass and microenvironment, and their function might be important for tumor progression in terms of diverse aspects such as angiogenesis, tumor cell invasion, motility, survival, and premetastatic niche establishment [8,21,22,37]. In this context, they are known as TAMs and present suppressive characteristics, some of which are like those of the M2 phenotype [25]. Given their phenotypic plasticity and dynamic function, it is indispensable to understand their generation and possible modulators in the tumoral context. Therefore, this work was developed to investigate how tumor-soluble product can interfere with macrophage development from bone marrow precursor cells until polarization. For this, products released by two tumor murine cell lines (3LL or MN/MCA) were used as stimuli along with M-CSF to obtain macrophage differentiation. Polarization was stimulated using IFN-γ and LPS (M1) or IL-4 (M2).

Tracking possible alterations in their development, we started with a phenotypic analysis which showed no differences between tumor-conditioned and control macrophage F4/80 expression, with around 90% of F4/80^+^ cells at day 7. This result pointed to a possible macrophage differentiation inducer present in tumor CM, since they were capable of stimulating macrophage development alone. Surprisingly, the quantification of M-CSF within the tumor supernatants showed the presence of approximately 25 pg/mL (or absence in some 3LL CM), a low concentration compared to the effect on F4/80 expression.

Following the polarization, we identified that Arg1 was highly expressed by cells differentiated with MN/MCA supernatant and activated to M2. Arginase, an enzyme that metabolizes L-arginine into L-ornithine and urea, is involved in several inflammatory events, including cancer [38]. Alternatively, L-arginine may be a substrate of nitric oxide synthase (NOS), whose expression is drastically increased in M1 macrophages and is responsible for the production of NO, a highly cytotoxic substance [39]. In our experiments, despite being similar to the expression of Nos2 by the M1 control and M1 conditioned with MN/MCA supernatant, Arg1 was strongly increased in M1 when MN/MCA products were present. In M0 and M2 polarized cells, MN/MCA CM was still capable of enhancing Arg1 expression, suggesting that NO synthesis is essentially impaired. In contrast, Fizz1, whose expression is well characterized in helminth infection, was diminished by MN/MCA CM in M2-like cells, demonstrating that not all classical M2-markers are positively modulated by the tumor products.

The impairment of IFN-β and TGF-β mRNA expression by MN/MCA CM is also portrayed here. Lu and co-workers reported in 2019 [40] that type I interferons, which include IFN-α and IFN-β, suppress tumor growth by boosting cytotoxic T lymphocytes in the tumor microenvironment. Colorectal samples from cancer patients display a reduced expression of IFN receptor 1 (IFNAR1) as compared to healthy samples. Moreover, tumors grow faster in animals that lack IFNAR1, specifically in T lymphocytes [40]. Whilst IFN type I mostly acts in controlling tumor development, TGF-β presents a paradoxical feature. TGF-β can induce a proangiogenic environment and stimulate tumor-related angiogenesis in certain tumor types [41]. However, its angiostatic function has also been demonstrated in pancreatic and gastric cancers via the induction of thrombospondin 1, a potent angiogenic inhibitor producer [42]. Proliferation, apoptosis, and differentiation may also be controlled by TGF-β, as demonstrated by Wang et al. [43], who showed the induction of autophagy of hepatocellular carcinoma cells by TGF-β signaling.

The microRNA miR-155 is typically expressed at high levels by M1 cells, but its expression was reduced by MN/MCA products in M1-like macrophages. Ye et al. [44] reported that miR-155 inhibition decreases the release of proinflammatory cytokines such as IL-6 and TNF-α through upregulation of the transcription factor suppressor of cytokine signaling 1 (SOCS1) and downregulation of signal transducer and activator of transcription 3 (STAT3) as well as of programmed cell death protein 4 (PDCD4). Moreover, it was shown that due to increasing miR-155 expression by TAMs from esophageal cancer, the expression of IL-12, TNF-α, and Nos2 are enhanced, and tumor cells survive and migrate less in this condition [45]. Therefore, miR-155 downregulation along with Arg1 upregulation observed in M1-like macrophages differentiated with MN/MCA CM might represent an advantage for tumor promotion.

Squadrito et al. [33] showed that miR-511-3p is expressed and active in both M2 and TAMs, and its increased expression is associated with CD206 expression. However, the overexpression of miR-511-3p inhibited tumor growth but did not affect the proinflammatory characteristic of their cells. In the present work, we did not observe CD206 modulation by tumor CM (data not shown), but miR-511 expression was increased in all conditions with MN/MCA CM and mostly with 3LL CM. MiR-511 has been correlated with both the induction and prevention of cell proliferation in different tumor settings. Zhang et al. [46] reported that overexpression of miR-511 promoted hepatocellular carcinoma proliferation by targeting B cell translocation gene 1 (BTG1). However, in a model of osteosarcoma it was demonstrated that miR-511 transfection resulted in lower expression of mitogen-activated protein kinase 1 (MAPK1) which inhibited osteosarcoma MG63 cell proliferation and invasion [47]. Nevertheless, there is no evidence for a role of miR-511 in macrophage proliferation.

The quantities of macrophages derived from bone marrow progenitors, from circulating monocytes, or from tissue-resident self-renewal varies according to the organ and to the presence of any pathology. For instance, in the skin and intestine most macrophages originate from monocyte differentiation even in a steady state [48,49], while in most other tissues the self-renewal of resident macrophages seems to be the major mechanism for the maintenance of their number [50]. Even though macrophage proliferation in the tumor environment has not been deeply explored, it is now established that mature macrophages, especially M2, are able to proliferate in situ, allowing their self-renewal independent of progenitor recruitment [51,52]. Giurisato and co-workers [53] showed that extracellular signal-regulated kinase 5 (ERK5) expression is required in macrophages under proliferation in a model of tumor grafts in mice and its deficiency in myeloid cells negatively modulated macrophage proliferation in metastatic tissues. Moreover, ERK5 maintains cell proliferation by suppressing p21 expression, thus affecting macrophage differentiation [53]. Here, we reported that about 5% of M2 polarized macrophages proliferated (data not shown), unlike M1 macrophages. This proliferation index seemed to be increased by 3LL and MN/MCA supernatants when cells on the fourth day of culture were analyzed. Macrophage proliferation in response to tumor CM might be not completely dependent on M-CSF/CD115 signaling, since we could not observe the complete reversion of proliferation when anti-CD115 was added. The low concentration of M-CSF in the tumor CM also corroborates this hypothesis, and the effects seen with tumor CM alone could be due to another M-CSF receptor ligand (e.g., IL-34).

On the other hand, a preferential expansion of CD16^+^ macrophages was observed. This might be of relevance as this population is characterized by an M2-like phenotype, as indicated by their ability to mediate fibrotic events in inflammatory diseases [54]. CD14^+^CD16^+^ monocytes were also described to produce high levels of IL-10 and to contribute to angiogenesis [55,56]. In conclusion, MN/MCA products are stronger modulators of macrophages than 3LL, and most of the effects observed were obtained in cells polarized into M2. Our hypothesis is that tumor products may act by preparing cells and making them more susceptible to IL-4. The fact that bone marrow-derived cells survive and can proliferate in the presence of tumor supernatants, even in the absence of exogenous M-CSF, is consistent with their abundance within the tumor microenvironment [15,21,22,23]. Therefore, we provide evidence that tumor-secreted products not only induce macrophage differentiation but also act to promote the survival and proliferation of macrophages with some protumoral features.

## Figures and Tables

**Table 1:** Percentage of F4/80^+^ cells after differentiation and polarization of bone marrow precursor cells (day 7). Data show media ± SD of 4 independent experiments.

	% F4/80^+^ Cells (Media ± SD)
M-CSF	95.64 ± 4.79
M-CSF + 3LL CM	96.77 ± 2.87
M-CSF + MN/MCA CM	94.96 ± 6.51
M-CSF (IFN-γ + LPS)	97.16 ± 2.52
M-CSF + 3LL CM (IFN-γ + LPS)	96.91 ± 2.65
M-CSF + MN/MCA CM (IFN-γ + LPS)	97.21 ± 2.68
M-CSF (IL-4)	96.80 ± 2.69
M-CSF + 3LL CM (IL-4)	97.61 ± 1.97
M-CSF + MN/MCA CM (IL-4)	97.16 ± 4.20
3LL CM	92.85 ± 6.43
MN/MCA CM	94.74 ± 6.33

## Data Availability

The data presented in this study are available on request from the corresponding author.
